# Shaping the Future of Dentistry: How Digital VR-Haptic Thinkers Are Revolutionizing Education by Thinking Big for Better Future in Oral Care

**DOI:** 10.1055/s-0045-1810611

**Published:** 2025-08-18

**Authors:** Reinhard Chun Wang Chau, Manchorova Neshka, Mihaela Pantea, Sarah Rampf, Mikko Liukkonen, David P. Rice, David Morton, Octave Nadile Bandiaky, Masako Nagasawa, Kinga Bágyi, Barry F. Quinn, Esther Carramolino-Cuéllar, Sónnica Galán-Gil, Nicla Flacco, Damiano Pasqualini, Muhammad A. Shazib, Simona-Georgiana Schick, Bekhzod Yarmukhamedov, Kristin Ackerman, Santiago Arias-Herrera, Ulla-E Palotie, Ahmed Adam Kada, Nawal Bouyahyaoui, Edgar Quenta-Silva, Gitana Rederiene, Nisrine El Arrouf, María P. Rodríguez-Hopp, Anna L. Suominen, Hannelie Edgar, Nicola Shanks, Amanda Jackson, Brid Hendron, Sila Nur Usta, Peter Lingström, Ulf Örtengren, Margrit Maggio, Niku Sondagar, Maxstein M. Abuzaid, Małgorzata Ponto-Wolska, Walter Yu Hang Lam, Piotr A. Regulski, Outi Huhtela, Łukasz Zadrożny, Mengwei Pang, Suzie Bergman, Szabolcs Felszeghy, Sompop Bencharit, Maria F. Sittoni-Pino

**Affiliations:** 1 Division of Restorative Dental Sciences, Faculty of Dentistry, The University of Hong Kong, Hong Kong, Hong Kong; 2 Department of Operative Dentistry and Endodontics, Faculty of Dental Medicine, Medical University of Plovdiv, Plovdiv, Bulgaria; 3 Department of Fixed Prosthodontics and Occlusology, Faculty of Dentistry, “Carol Davila” University of Medicine and Pharmacy, Bucharest, Romania; 4 Department of Conservative Dentistry, Clinic for Oral, Dental and Maxillofacial Diseases, Heidelberg Medical Faculty, Heidelberg University, Heidelberg, Germany; 5 Division of Ophthalmology, Institute of Clinical Medicine, School of Medicine, University of Eastern Finland, Kuopio, Finland; 6 Department of Oral and Maxillofacial Diseases, Faculty of Medicine, University of Helsinki and Helsinki University Hospital, Finland; 7 Department of Neurobiology, School of Medicine, University of Utah, Salt Lake City, Utah, United States; 8 Division of Regenerative Medicine and Skeleton, Faculty of Dentistry, University of Nantes, Nantes, France; 9 Division of Bio-Prosthodontics, Faculty of Dentistry and Graduate School of Medical and Dental Sciences, Niigata University, Niigata, Japan; 10 Department of Operative Dentistry and Endodontics, Faculty of Dentistry, University of Debrecen, Debrecen, Hungary; 11 Department of Restorative Dentistry, School of Dentistry, Faculty of Health and Life Sciences, Institute of Life Course and Medical Sciences, University of Liverpool, Liverpool, United Kingdom; 12 Department of Odontology, Faculty of Health Sciences, Universidad Europea de Valencia, Valencia, Spain; 13 Department of Surgical Sciences, Dental School, University of Turin, Turin, Italy; 14 Department of Dental Medicine, Workman School of Dental Medicine, High Point University, High Point, North Carolina, United States; 15 Tashkent State Dental Institute, Tashkent, Uzbekistan; 16 Dental Medicine Faculty, Mohammed V. University in Rabat, Rabat, Morocco; 17 Stomatology Simulation Center, Facultad de Estomatología, Universidad Peruana Cayetano, Heredia, Lima, Peru; 18 European Dental Hygienists Federation, Utrecht, Netherlands; 19 Facultad de Salud y Odontología, Universidad Diego Portales, Santiago, Chile; 20 Oral Health Teaching Unit, Kuopio University Hospital, Kuopio, Finland; 21 Northern Ireland Medical and Dental Training Agency (NIMDTA), Belfast, Northern Ireland, United Kingdom; 22 Department of Restorative Dentistry & Endodontics, Gulhane Faculty of Dentistry, University of Health Sciences Ankara, Ankara, Türkiye; 23 Department of Cariology, Institute of Odontology, Sahlgrenska Academy, University of Gothenburg, Gothenburg, Sweden; 24 Note Dr Ltd, Covent Garden, London, United Kingdom; 25 Department of Propaedeutics and Dental Prophylaxis, Faculty of Medicine and Dentistry, Warsaw Medical University, Warsaw, Poland; 26 Institute of Dentistry, School of Medicine, University of Eastern Finland, Kuopio, Finland; 27 Department of Prosthodontics, School of Stomatology, Chongqing Medical University, Chongqing, China; 28 Department of Neurosurgery, Oregon Health and Science University, Multnomah County, Oregon, United States

**Keywords:** dental education, virtual reality, haptic technologies, digital, artificial intelligence, psychomotor skills, curriculum integration

## Abstract

Integrating digital, virtual reality (VR), and haptic technologies into dental education transforms training by fostering immersive, student-centered environments. These tools bridge theoretical knowledge and clinical practice, enhancing psychomotor skills, reducing anxiety, and enabling standardized assessments in risk-free settings. The Digital, VR, and Haptic Thinkers Network drives global collaboration and innovation. Drawing on insights from a 2025 conference at Queen Mary University of London, this commentary explores advancements, challenges, and future directions for VR-haptic integration in dental curricula. Results from global implementations, including improved manual dexterity and procedural precision, as well as enhanced pulpotomy performance, demonstrate a significant educational benefit. In clinical dentistry, VR-haptic simulators enhance patient outcomes by facilitating precise skill development for procedures in restorative dentistry and endodontics, thereby reducing errors in real-world practice. Combining these technologies with traditional methods is proposed to prepare competent, confident dental professionals while addressing educational disparities. Rigorous validation, strategic curriculum integration, and interdisciplinary collaboration are recommended to maximize impact.

## Introduction

Dental education faces persistent challenges: limited clinical training opportunities, student anxiety, and inconsistent skill assessments. 1 These issues are particularly pressing in Europe and North America, where the rising demand for dental care is projected to increase due to aging populations and heightened oral health awareness. This strain on dental health care and educational systems calls for stronger primary oral health care and innovation in dental education. 2 3 Globally, disparities in access to training exacerbate inequities in oral health care delivery, particularly in underserved regions.

Digital, virtual reality (VR), and haptic technologies offer transformative solutions by providing immersive, tactile learning environments that replicate real-world procedures without exposing patients to risk. 4 These tools enhance precision, confidence, ergonomic awareness, and long-term knowledge retention, addressing gaps in traditional training. 5 VR and haptic systems are now widely adopted across several medical disciplines, including surgery, 6 dentistry, 5 anesthesiology, 7 gynecology, 8 orthopedics, 9 and rehabilitation. 10 In surgery and dentistry, for example, haptic simulators support the development of essential manual skills, while in rehabilitation, they aid sensorimotor recovery. These technologies provide learners with the opportunity to practice procedures in safe, controlled environments, where tactile feedback enhances the realism and effectiveness of skill acquisition. Recent research highlights the significant potential of VR-haptics to complement traditional training methods, offering individualized feedback and boosting learner engagement. However, ongoing validation and research are needed to fully establish these tools as primary training modalities in dental and medical education.

The global relevance of this topic is underscored by the Digital, VR, and Haptic Thinkers Consortium, established in 2023, which unites educators, researchers, and industry leaders to advance these technologies. Insights from its 2025 conference at Queen Mary University of London (QMUL) highlight the potential of this approach to reshape dental education (see Appendix 1 , available in the online version only). This commentary explores how VR-haptic technologies can address educational challenges, offering a perspective informed by global research and practical implementations. By integrating these tools, dental education can prepare professionals to meet the demands of modern health care while promoting equitable access to training. As technology advances, VR and haptics are poised to play an increasingly central role in both learning and assessment in dental education.

## VR-Haptic Technologies in Dental Education

### Current Advancements

#### Immersive Skill Development

VR-haptic simulators, such as the Simodont Dental Trainer and SIMtoCARE, deliver realistic tactile feedback, enabling students to refine their psychomotor skills in controlled settings. 11 Since their installation at the University of Eastern Finland in 2021, these simulators have improved the manual dexterity and procedural precision of dental students. Similarly, Universidad Europea de Valencia's integration of VR-haptics into pediatric dentistry since 2019 has enhanced pulpotomy performance through tailored simulations. These tools shorten learning curves, with studies showing improved technical proficiency and patient care outcomes. VR-haptics also supports skill maintenance for experienced practitioners, ensuring lifelong learning. 12

#### Standardized Assessment and Feedback

VR-haptic systems provide objective, real-time performance evaluations, reducing subjectivity in traditional assessments. A survey at the Medical University of Warsaw found that 60% of students valued instant feedback, with 80% reporting improved learning outcomes. 13 Blended approaches—combining automated system evaluations with educator feedback—are preferred by 70% of students, fostering a balanced learning experience. Such systems enable consistent, scalable assessments for standardizing training across diverse global institutions.

#### Reducing Anxiety and Enhancing Well-Being

VR-haptic technologies significantly alleviate student anxiety by providing safe, controlled environments for skill development, allowing mistakes without real-world consequences. 11 Simulations enable gradual exposure to complex procedures, such as cavity preparation or root canal therapy, fostering confidence through repetitive practice. 14 For example, previous studies found that students performed 30% faster after training in simulated clinical scenarios 11 15 and showed improvement in anesthesia accuracy by up to 84%, 16 with 75% students reporting support for inclusion of VR/haptics/artificial intelligence (AI) in curricula. 15 17 This is particularly relevant for Generation Z students, whose fine motor skills may be underdeveloped due to reliance on digital devices. 18 VR-haptics bridge the gap between digital familiarity and clinical competence, enhancing psychomotor skills in a low-pressure setting, where students can practice without fear of irreversible errors, boosting confidence and well-being. 12 15

Moreover, VR-haptic environments promote psychological well-being by incorporating gamification elements, such as progress tracking and achievement milestones, which make learning an engaging and rewarding experience. 19 Studies have reported an increase in motivation and a reduction in burnout among students using gamified VR-haptic modules compared to traditional training cohorts. 12 20 These systems also support personalized learning paces, accommodating diverse learner needs and reducing performance-related stress. 11 21 By fostering a supportive, mistake-tolerant environment, VR-haptics empower students to build resilience and confidence, critical for their long-term professional well-being. 12 17

#### Global Collaboration and Accessibility

Initiatives like the 2025 QMUL conference, supported by industry leaders like Dentsply Sirona and the American Dental Education Association, foster global collaboration. AI-powered VR tools and mobile health (mHealth) technologies may empower clinicians to deliver care to underserved populations, addressing disparities in oral health care access. 22 23 These efforts promote equitable access, aligning with global health priorities.

### Limitations and Challenges in Adoption

Despite their promise, VR-haptic and AI technologies face significant limitations in dental education. High initial costs deter adoption, particularly in resource-limited settings, where financial constraints restrict access to advanced simulators. 4 Resistance to change among educators, often stemming from unfamiliarity with digital tools or concerns about diminishing the role of traditional methods, complicates implementation. 5 Logistical challenges, such as integrating these tools into existing curricula and ensuring compatibility with diverse education systems, require meticulous planning and faculty training. 12 Additionally, the lack of universal standards for VR-haptic training protocols can lead to inconsistent quality and outcomes across institutions. 24 Rigorous validation studies are necessary to confirm the efficacy of new methods compared to traditional ones, ensuring evidence-based adoption. 12 25 For instance, studies evaluating VR, haptics, and AI technologies underscore the importance of validating these digital tools to ensure their reliability and effectiveness in dental educational settings. 15 26 Ethical considerations, particularly regarding equitable access for underserved regions, must also be addressed to prevent widening disparities. 12 27

### Perspective: A Balanced Approach to Transformation

The authors advocate a balanced approach, integrating VR-haptic technologies with traditional methods to optimize skill acquisition. 28 This perspective is grounded in global research and practical insights from institutions like the University of Eastern Finland and Universidad Europea de Valencia. VR-haptics excel in providing risk-free, immersive training; however, hands-on practice with typodonts and phantom heads remains essential for achieving tactile realism. Blended learning environments, combining digital and physical training, maximize technical proficiency and confidence. 4

This perspective emphasizes strategic curriculum integration, prioritizing early practical experience to develop psychomotor and attitudinal competencies. The Specific, Measurable, Achievable, Relevant, and Time-Bound (SMART) model offers a framework for implementation, ensuring clear objectives and measurable outcomes. AI integration enhances VR-haptics by providing personalized feedback and predictive analytics, further shortening learning curves. 12 Global standardization of VR-haptic training and credentialing is critical to ensure equitable access and consistent quality across institutions.

Collaboration is central to this vision. The Digital, VR, and Haptic Thinkers Network exemplifies interdisciplinary efforts, uniting academia, industry, and professional bodies. By fostering innovation and sharing best practices, such networks can overcome financial, logistical, and cultural barriers. Ethical frameworks are crucial for prioritizing patient-centered outcomes and ensuring equitable access, particularly among underserved regions and populations. 27

## Summary and Recommendations

VR-haptic technologies hold transformative potential for dental education, addressing critical challenges in skill development, assessment, and student well-being. 4 Their ability to create immersive, risk-free training environments enhances technical proficiency and promotes equitable education access. 11 As dental care demands rise globally, these tools are vital for preparing competent and confident professionals. However, financial constraints, resistance to change, and the need for validation studies hinder adoption. 17

To realize this potential, the authors recommend:

- Strategic curriculum integration: adopt the SMART model to integrate VR-haptics transversally, prioritizing early practical experience and blended learning approaches.
- Increased investment: fund validation studies, educator training, and infrastructure, particularly in resource-limited settings, to ensure evidence-based adoption.
- Global standardization: develop universal VR-haptic training and credentialing standards to ensure equitable access and consistent quality.
- Interdisciplinary collaboration: strengthen networks like the Digital, VR, and Haptic Thinkers Network to foster innovation and share best practices.
- Ethical frameworks: establish guidelines to prioritize patient-centered outcomes and equitable access, aligning with global health priorities.

By implementing these recommendations, dental education can evolve to meet modern health care demands, ensuring graduates are equipped to deliver high-quality care. 28 Combined with traditional methods, VR-haptic technologies offer a path to reimagine how dentistry is taught, fostering a future where education is immersive, inclusive, and impactful. 4

## Notes

**Corrigendum** : The article has been updated as per Corrigendum published on December 22, 2025 (DOI: 10.1055/s-0045-1814167).
