# Corrigendum: Shaping the Future of Dentistry: How Digital VR-Haptic Thinkers Are Revolutionizing Education by Thinking Big for Better Future in Oral Care

**DOI:** 10.1055/s-0045-1814167

**Published:** 2025-12-23

**Authors:** Reinhard Chun Wang Chau, Manchorova Neshka, Mihaela Pantea, Sarah Rampf, Mikko Liukkonen, David P. Rice, David Morton, Octave Nadile Bandiaky, Masako Nagasawa, Kinga Bágyi, Barry F. Quinn, Esther Carramolino-Cuéllar, Sónnica Galán-Gil, Nicla Flacco, Damiano Pasqualini, Muhammad A. Shazib, Simona-Georgiana Schick, Bekhzod Yarmukhamedov, Kristin Ackerman, Santiago Arias-Herrera, Ulla-E Palotie, Ahmed Adam Kada, Nawal Bouyahyaoui, Edgar Quenta-Silva, Gitana Rederiene, Nisrine El Arrouf, María P. Rodríguez-Hopp, Anna L. Suominen, Hannelie Edgar, Nicola Shanks, Amanda Jackson, Brid Hendron, Sila Nur Usta, Peter Lingström, Ulf Örtengren, Margrit Maggio, Niku Sondagar, Maxstein M. Abuzaid, Małgorzata Ponto-Wolska, Walter Yu Hang Lam, Piotr A. Regulski, Outi Huhtela, Łukasz Zadrożny, Mengwei Pang, Suzie Bergman, Szabolcs Felszeghy, Sompop Bencharit, Maria F. Sittoni-Pino

**Affiliations:** 1 Division of Restorative Dental Sciences, Faculty of Dentistry, The University of Hong Kong, Hong Kong, Hong Kong; 2 Department of Operative Dentistry and Endodontics, Faculty of Dental Medicine, Medical University of Plovdiv, Plovdiv, Bulgaria; 3 Department of Fixed Prosthodontics and Occlusology, Faculty of Dentistry, “Carol Davila” University of Medicine and Pharmacy, Bucharest, Romania; 4 Department of Conservative Dentistry, Clinic for Oral, Dental and Maxillofacial Diseases, Heidelberg Medical Faculty, Heidelberg University, Heidelberg, Germany; 5 Division of Ophthalmology, Institute of Clinical Medicine, School of Medicine, University of Eastern Finland, Kuopio, Finland; 6 Department of Oral and Maxillofacial Diseases, Faculty of Medicine, University of Helsinki and Helsinki University Hospital, Finland; 7 Department of Neurobiology, School of Medicine, University of Utah, Salt Lake City, Utah, United States; 8 Division of Regenerative Medicine and Skeleton, Faculty of Dentistry, University of Nantes, Nantes, France; 9 Division of Bio-Prosthodontics, Faculty of Dentistry and Graduate School of Medical and Dental Sciences, Niigata University, Niigata, Japan; 10 Department of Operative Dentistry and Endodontics, Faculty of Dentistry, University of Debrecen, Debrecen, Hungary; 11 Department of Restorative Dentistry, School of Dentistry, Faculty of Health and Life Sciences, Institute of Life Course and Medical Sciences, University of Liverpool, Liverpool, United Kingdom; 12 Department of Odontology, Faculty of Health Sciences, Universidad Europea de Valencia, Valencia, Spain; 13 Department of Surgical Sciences, Dental School, University of Turin, Turin, Italy; 14 Department of Dental Medicine, Workman School of Dental Medicine, High Point University, High Point, North Carolina, United States; 15 Tashkent State Dental Institute, Tashkent, Uzbekistan; 16 Dental Medicine Faculty, Mohammed V. University in Rabat, Rabat, Morocco; 17 Stomatology Simulation Center, Facultad de Estomatología, Universidad Peruana Cayetano, Heredia, Lima, Peru; 18 European Dental Hygienists Federation, Utrecht, Netherlands; 19 Facultad de Salud y Odontología, Universidad Diego Portales, Santiago, Chile; 20 Oral Health Teaching Unit, Kuopio University Hospital, Kuopio, Finland; 21 Northern Ireland Medical and Dental Training Agency (NIMDTA), Belfast, Northern Ireland, United Kingdom; 22 Department of Restorative Dentistry & Endodontics, Gulhane Faculty of Dentistry, University of Health Sciences Ankara, Ankara, Türkiye; 23 Department of Cariology, Institute of Odontology, Sahlgrenska Academy, University of Gothenburg, Gothenburg, Sweden; 24 Note Dr Ltd, Covent Garden, London, United Kingdom; 25 Department of Propaedeutics and Dental Prophylaxis, Faculty of Medicine and Dentistry, Warsaw Medical University, Warsaw, Poland; 26 Institute of Dentistry, School of Medicine, University of Eastern Finland, Kuopio, Finland; 27 Department of Prosthodontics, School of Stomatology, Chongqing Medical University, Chongqing, China; 28 Department of Neurosurgery, Oregon Health and Science University, Multnomah County, Oregon, United States

The above article, published eFirst in the *European Journal of Dentistry* in August 2025 (doi: 10.1055/s-0045-1810611 ), has been updated to reflect the correct name of its sixth author, David P. Rice. It was previously listed incorrectly as David R. Rice. The article has now been corrected accordingly.

