# Supplementary material for: Shaping the Future of Dentistry: How Digital VR-Haptic Thinkers Are Revolutionizing Education by Thinking Big for Better Future in Oral Care
Source: Eur J Dent. 2025 Aug 18;20(2):669–72. doi: 10.1055/s-0045-1810611 (PMC13160611; doi:10.1055/s-0045-1810611)
Supplement: Supplementary file 1 — Supplementary Material [file 10-1055-s-0045-1810611-s2554290.pdf]

Appendix 1: Quotes from each of the authors

1. "We need to dare to think big. By developing VR-haptics-based training programs across various dental courses and locations, we hope to provide students with broader access to advanced resources for diverse dental procedures. When we launched the original VR-Haptic Thinkers group in 2023, our primary objective was to foster a collaborative environment that would harness the transformative potential of digital, VR, and haptic technologies in dental education."—Liisa Suominen
2. "After receiving the equipment (VR-haptic Simodont® Dental Trainers (Nissin)), we immediately decided to begin researching their use."—Outi Huhtela
3. "Integrating digital, AI, and haptics-reinforced, patient-specific VR technologies will enhance dental students' technical proficiency, reduce learning curves, and improve patient care outcomes while unifying education globally. By combining VR-haptic feedback with real-world exercises, CADCAMs, and 3D printing, dental educators can create a holistic training environment that prepares students for clinical excellence and addresses the cognitive and emotional challenges of modern learners. This dual approach is essential for shaping confident, skilled surgeons who are ready to meet the demands of tomorrow's healthcare landscape."—Sompop Bencharit
4. "VR-haptic technology enhances dental hygiene education by providing realistic tactile feedback, allowing dental hygiene students to develop precise psychomotor skills in a safe, simulated environment. It enables mistake-based learning and repeated practice without risk to patients, fostering confidence and clinical readiness. These tools bridge the gap between theory and practice by reinforcing anatomical knowledge through interactive, hands-on experiences. Educators benefit from standardized, measurable assessments that objectively track dental hygiene student progress. Overall, VR-haptic thinking transforms traditional training, ensuring more consistent, practical, and relevant preparation for real-world dental hygienist practices in different regions."—Gitana Rederiene
5. "VR-haptics serves as more than just a technical tool; it is a 'Dr. X' for dentistry—a comprehensive trainer for skill development, an ergonomics coach, and even a psychotherapeutic aid. To fully realize its potential, this cutting-edge technology must be complemented by hands-on activities that reinforce tactile learning and manual proficiency."—Szabolcs Felszeghy
6. "Digital technologies offer transformative benefits in healthcare education, but their adoption is not without hurdles. The high initial investment required can be prohibitive, especially for institutions in resource-limited settings. Resistance to change among educators accustomed to traditional methods further complicates integration efforts."—David R. Rice
7. "Digital tools have immense potential to enhance visualization, objective assessment, stress reduction, and accelerated learning in dental education. Addressing cost barriers, resistance, integration complexities, and validation concerns will be key to unlocking their full potential."—Ulla-E Palotie
8. "Nissin drilling plates offer a promising dental hard tissue handling simulation, particularly for handpiece-naïve students in preclinical dental education. With minor refinements and considering the challenges of haptically reproducing dental tissues, these tools could seamlessly integrate into existing curricula, representing a valuable addition to training methodologies and fostering confident, skilled professionals."—Sarah Rampf
9. "Virtual haptic simulators are game-changing in endodontic education, as they can refine procedural precision while alleviating student anxiety."—Sıla N. Usta
10. "Artificial intelligence (AI) and mobile health (mHealth) are transforming community dentistry. AI tools can empower students to deliver impactful oral health care to underserved populations. Blending these technologies with traditional training equips future dentists to address oral health inequities effectively."—Reinhard C. W. Chau
11. "Artificial Intelligence is revolutionizing surgical training by providing real-time, personalized feedback that enhances skill development and ensures patient safety. By analyzing vast amounts of surgical data, AI can objectively assess technical abilities, predict outcomes, and identify areas for improvement. This technology shortens trainees' learning curve and optimizes surgical performance through tailored guidance and actionable insights. As we integrate AI into training programs, we are paving the way for a more efficient, precise, and patient-centered approach to surgery."—Niku Sondagar
12. "Modern dental education demands rigorous training to master surgical techniques. VR haptic simulation offers a compelling solution, enhancing skills in a risk-free environment and allowing for unlimited practice and refinement, ultimately minimizing potential harm to patients."—Damiano Pasqualini
13. "As educators, we cannot forget that Digital technologies with AI, VR, and Haptic simulators were developed for students, and their feedback regarding training and developing motoric skills and practical competences is crucial for the shape of future Dental Education."—Łukasz Zadrożny
14. "With VR glasses, students may train and practice correct order, e.g., for adhesive protocols in restorative dentistry, any other protocol used in dental clinics."—Małgorzata Ponto-Wolska and Piotr Regulski
15. "This is not just about technology—it's about reimagining how we teach and learn."—Simona-Georgiana Schick
16. "The integration of digital technologies must prioritize early practical experience to support the development of psychomotor skills, as well as attitudinal competencies that reinforce patient-centered educational models—such as teamwork, critical thinking, and effective communication."—María P. R. Hopp

 © 2025. The Author(s).

17. “I firmly believe that integrating advanced technologies into dental education is essential for bridging the gap between theoretical knowledge and practical skills. However, the implementation of such systems goes beyond strategic planning—it demands significant resources, including physical spaces, educator training, and overcoming resistance to change.”—Kinga Bagyi
18. “Educators often question, ’Why fix something that isn’t broken?’ As leaders, we must clearly envision how these digital tools will enhance specific parts of our curriculum. Without this foresight, technology risks becoming an unused relic, gathering dust like a forgotten artifact. Our focus must remain on ensuring that these innovations actively contribute to the development of future dental professionals.”—Ali Schazib
19. “VR-haptic technology is not just a tool but a bridge between theoretical knowledge and clinical excellence. Allowing students to practice unlimited times in a stress-free environment builds confidence and ensures precision in procedures like cavity preparation and drilling. This technology provides immediate feedback, enabling learners to refine their techniques effectively while minimizing errors—a crucial factor for both education and patient safety.”—Peter Lingström
20. “Digital technologies, such as virtual reality and haptics, are revolutionizing how students learn and practice dentistry. These tools provide immersive experiences that enhance skill acquisition and promote well-being by reducing stress and anxiety and boosting confidence. Importantly, they positively influence networking, as well. Through digital initiatives, we are integrating these advancements to empower aspiring dentists and prepare the next generation for success in a rapidly evolving field where physical and mental well-being are paramount.”—Suzie Bergman
21. “Moreover, using such technologies transcends technical skill acquisition. In anatomy education, for example, dissection is not just about learning structures; it connects students to the profound realities of life and death, cultivating empathy, professionalism, and respect for donors. This holistic approach prepares future healthcare professionals to deliver compassionate and humane patient care. On a practical level, dissection bridges the gap between theory and clinical practice. It provides an irreplaceable three-dimensional understanding of anatomical structures and spatial relationships, critical for surgical precision and diagnostic accuracy. Handling real tissues builds manual dexterity and confidence in ways no digital simulation can fully replicate.”—David Morton
22. “The first subject to introduce VR haptics exercises in preclinical practices (Universidad Europea de Valencia, Spain) was Pediatric Dentistry. with the addition to the course’s curricula of customized pulpotomy cases based on competency goals and student experience level, VR haptic simulators offered students the possibility to practice pulpotomy key procedural steps, including assessing caries extent, creating precise pulp chamber openings, and removing affected pulp tissue.”—Sittoni-Pino
23. “VR & Haptic training combined with traditional phantom heads practice significantly improves students’ manual dexterity and spatial awareness.”—Anabel Gramatges-Rojas
24. “The application of swLORETA qEEG is a cutting-edge approach characterized by advanced temporal resolution compared to fMRI, low cost, and harmlessness. It illuminates and clarifies the intimate mechanisms of the formation of memory chains and introduces a new objective way for analyzing the effectiveness of learning, offers a way to validate the effects of learning on cognition, and detects in real time the impact of digital learning on cognitive modalities, including memory, visual-spatial discrimination, sensory-motor integration, and executive function.”—Manchorova, Kolev, and Kirkova-Bogdanova
25. “Excessive reliance on smartphones and computers has led to a decline in handwriting abilities among Gen Z, hindering the development of fine motor skills critical for prosthetic dental practice. This lack of manual dexterity often manifests as anxiety during practical training, underscoring the need for balanced educational strategies.”—Mihaela Pantea
26. “By allowing students to interact with highly detailed virtual patients, this technology enhances their confidence, technical precision, and adaptability in real clinical settings. In the future, such innovations could significantly improve the quality of education and patient care.”—Teodor R. Constantin
27. “Digital VR and haptic tools create a safe, controlled environment for students to practice essential skills without the fear of making irreversible mistakes on live patients.”—Masako Nagasawa
28. “Simulations allow learners to build confidence gradually, alleviating anxiety, often associated with successful clinical training. Incorporating digital tools early in training helps students develop proper ergonomic habits while reducing risks of musculoskeletal issues common in dentistry.”—Octave Bandiaky
29. “Haptics provide immersive simulations that enhance precision and allow students to practice repeatedly without fear of error, while typodonts offer tactile realism essential for developing manual dexterity. Together, they form a powerful synergy that equips students with the competence and confidence needed for clinical excellence.”—Anas Salim
30. “This method (integrating cutting-edge technology into clinical practice) improves precision and alignment and reduces chairside adjustments, ensuring a smoother patient experience. By integrating advanced digital tools, we can achieve faster, more predictable results while maintaining high-quality outcomes.”—Noha Barakat
31. “The ability to simulate intricate surgical steps ensures trainees are better prepared for real-world challenges,

 © 2025. The Author(s).

ultimately improving patient outcomes and advancing dental education. A dental alveolar surgery simulator and its innovative application suite have been developed and validated as a transformative tool for pre-clinical training. This cutting-edge technology offers dental students an invaluable opportunity to practice and perfect their skills in a safe, controlled environment before transitioning to real-life clinical settings. Beyond technical proficiency, this simulator can foster confidence and precision, empowering future dentists to approach patient care with competence and compassion. By bridging the gap between theory and practice, this advancement represents a significant step forward in shaping the next generation of skilled and empathetic dental professionals."—Mengwei Pang

32. "Virtual haptic simulation represents a significant leap forward in dental education. It not only allows students to refine their skills in a stress-free environment but also provides precise, objective, real-time (educator-free) evaluations of their performance."—Cristina M. Sanchez
33. "We explored integrating AI into serious games (SGs) for dental education, building on prior non-AI efforts. Using over 100 clinical cases, AI improved content creation, interactivity, and efficiency. Three bilingual SGs were developed, enhancing learning outcomes, motivation, and readiness."—Ahmed A. Kada
34. "Early ergonomic training is essential for reducing the risk of musculoskeletal disorders and ensuring long-term professional health. Blended learning with VR and haptics offers transformative possibilities for the future of education. By integrating immersive technologies, such as virtual reality and haptic feedback, learners can engage in realistic, multisensory simulations that enhance skill acquisition and spatial awareness across disciplines."—Stefan Danylak
35. "The first-ever global study on blended learning in dental education, conducted after the COVID-19 pandemic, marks a significant milestone in understanding educational practices across regions. Our findings underscore the need to tailor teaching strategies to meet students' preferences and learning needs better. This study sets the stage for future exploration into optimizing blended learning approaches for diverse educational contexts globally. By integrating standardized systems like motion sensors and video analysis, we can offer real-time feedback that helps students develop proper postural habits before tackling complex dental procedures. This might improve VR and haptic-supported blended learning and manual dexterity training in the future, as well."—Sobia Zafar
36. "Extended breaks can lead to a loss of confidence in performing clinical procedures. VR-haptic systems provide individualized training and real-time feedback, enabling users to identify and address specific areas for improvement."—Hannelie Edgar
37. "These (VR-haptic) systems allow users to practice at their own pace and convenience, making them ideal for professionals balancing family responsibilities with skill development."—Nicky Shanks
38. "Digital innovations - including haptic reinforced virtual reality systems - are revolutionizing dental education by creating standardized, immersive learning frameworks that transcend cultural and curricular boundaries. These technologies enable educators to design adaptable training modules rooted in universal clinical principles, fostering global collaboration through shared digital platforms. By harmonizing hands-on skill development and diagnostic methodologies across regions, we empower institutions to exchange best practices seamlessly, ensuring students worldwide receive equitable, cutting-edge training while preserving local educational values. This technological synergy not only bridges cross-cultural divides but also elevates leadership capabilities to cultivate a cohesive, future-ready global dental community."—Maxstein M. A. M. A. Mostafa

 © 2025. The Author(s).
